# Setting and prioritizing evidence-informed policies to control childhood obesity in Iran: a mixed Delphi and policy dialogue approach

**DOI:** 10.1186/s12887-022-03796-z

**Published:** 2022-12-20

**Authors:** Fatemeh Toorang, Amirhossein Takian, Hamed Pouraram, Parisa Amiri, Zahra Abdullahi

**Affiliations:** 1 Department of Community Nutrition, School of Nutritional Sciences and Dietetics, Tehran University of Medical Sciences, P.O. Box:1455-6119, Tehran, IR Iran; 2 Cancer Research Center, Cancer Institute, Tehran University of Medical Science, Tehran, Iran; 3 Departments of Global Health & Public Policy, School of Public Health, Tehran University of Medical Sciences, Tehran, Iran; 4 Department of Health Management, Policy and Economics, School of Public Health, Tehran University of Medical Sciences, P.O. Box: 1455-6119, Tehran, IR Iran; 5 Health Equity Research Center (HERC), Tehran University of Medical Sciences, Tehran, Iran; 6 Research Center for Social Determinants of Health, Research Institute for Endocrine Sciences, Shahid Beheshti University of Medical Sciences, Tehran, Iran; 7 Nutrition Office, Ministry of Health and Medical Education, Tehran, Iran

**Keywords:** Childhood obesity, Delphi, policy dialogue, evidenced-informed policy

## Abstract

**Background:**

The prevalence of childhood obesity (CO) and related complications is high and alarmingly increasing in Iran. This study applied a mixed Delphi & Policy Dialogue approach to exploring and prioritizing policy options to control childhood obesity in Iran.

**Methods:**

This study is organized in three Delphi phases followed by a policy dialogue session. This study applied the advocacy collation framework and evidence-informed policy-making approach to enhance the chance of a feasible and acceptable policy package. The first step consisted of interviews with 30 experts and primary stakeholders. Based on their answers and a comprehensive literature review, a list of presumed effective policy options to combat CO in Iran was made. Then, panelists were asked to score each policy option using a five-point Likert scale in seven constructs. To maximize the spread of opinions, panelists were chosen to represent three perspectives: policy-makers at different levels, presidents of various organizations who would implement potential policy options, and academics. Twenty-one stakeholders were invited to discuss the policy options in a policy dialogue section.

**Results:**

We introduced 27 policy options and asked stakeholders to rank them using seven criteria on a five-level Likert scale. Totally, 41 experts participated in round 2 (66.2% response rate), and 33 experts took part in round 3 (72% response rate). Participants believed that healthy schools, creating healthy environments in kindergartens and other child care centers, subsidizing healthy foods, educating healthy lifestyles in mass media, and increasing access to physical activity facilities are the most effective and feasible policies in controlling CO. After the policy dialogue, the healthy school remained the most prioritized policy. a policy package to combat CO in Iran was designed with the participation of all stakeholders.

**Conclusion:**

The advocacy collation framework and the evidence-informed policy-making approach were used to draft a policy package to combat CO, increasing the acceptability and feasibility of the developed policy package.

**Graphical Abstract:**

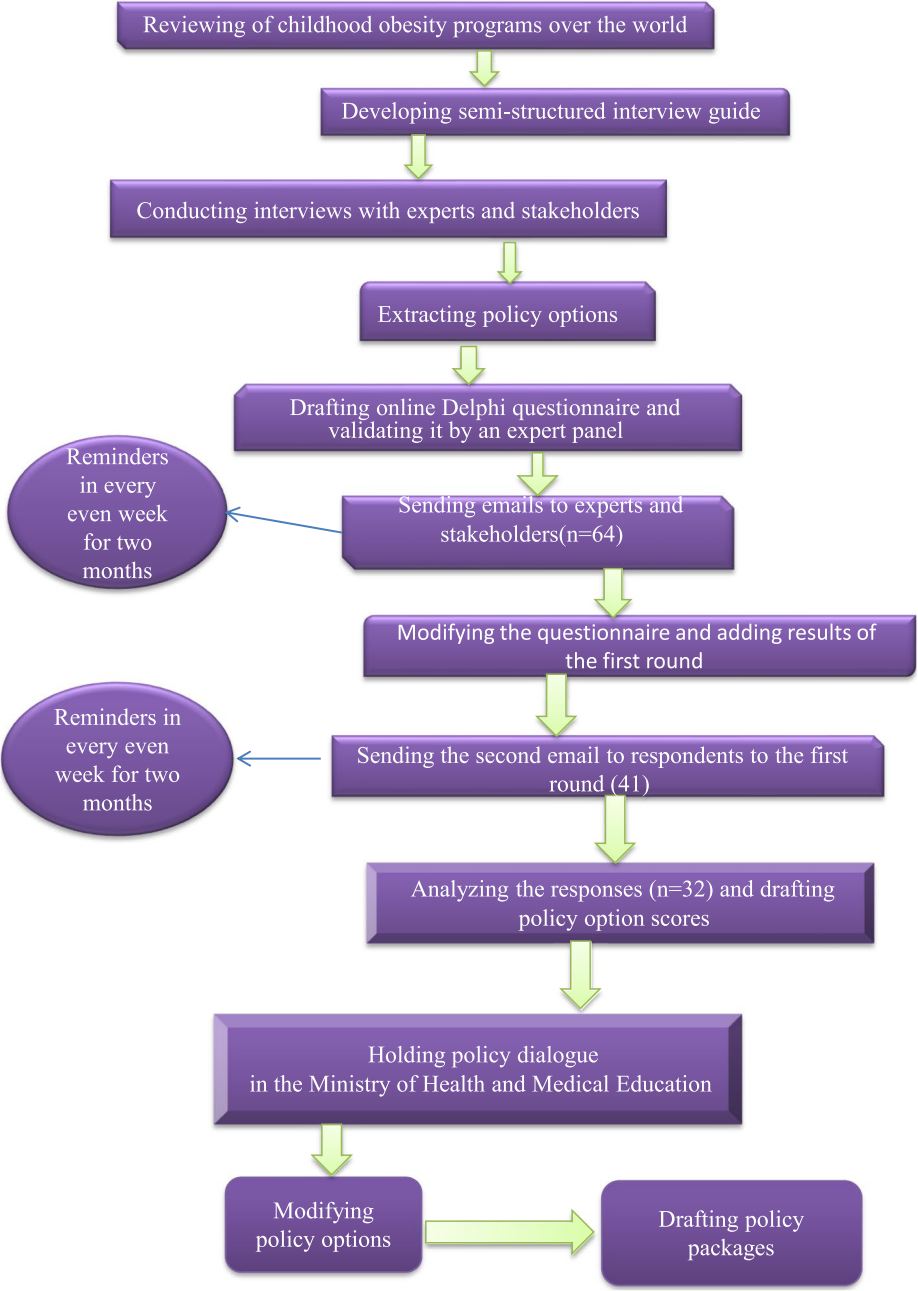

**Supplementary Information:**

The online version contains supplementary material available at 10.1186/s12887-022-03796-z.

## Highlights

Childhood obesity is a gateway to several diseases in the near and distant future.

The prevalence of childhood obesity is high and is increasing alarmingly in Iran.

Several policy options are proposed by scientists and other stakeholders to combat childhood obesity in Iran. Stakeholders believed that healthy schools, creating healthy environments in kindergartens and other child care centers, subsidizing healthy foods, educating healthy lifestyles in mass media, and increasing access to physical activity facilities with priority given to deprived areas are the most effective and feasible policies in controlling childhood obesity.

**Supplementary Information:**

The online version contains supplementary material available at 10.1186/s12887-022-03796-z.

## Background

Obesity and overweight are the gateways of non-communicable diseases like diabetes, cardiovascular and cerebrovascular diseases, several cancers, hypertension, hyperlipidemia, respiratory problems, arthritis, and other joint difficulties. Moreover, it has been proven that obesity disturbs the responses of the immunity system to several infections, including the covid-19 [1]. Several studies have demonstrated a mutual association between obesity and the covid-19 pandemic [2, 3].

Over the recent decade, a considerable surge in the prevalence of childhood obesity (CO) all over the globe has raised concerns about its subtractive effects on global health in the short and long run [4]. Early weight patterns play a significant role in lifelong weight trajectories, highlighting the importance of healthy childhood weight [5]. Being obese in 6 years is associated with a fourfold risk of being obese in adulthood [6, 7]. Children affected by obesity face many future health difficulties, including hypertension, type 2 diabetes, hyperlipidemia, asthma, sleep apnea, and psychological challenges [8]. Efforts to address obesity must significantly focus on early childhood, which could boost the primary and primordial prevention of several non-communicable diseases [5, 9].

Obesity is no longer merely a problem in high-income countries. Its burden increases worldwide, including in the Eastern Mediterranean Region (EMRO) [10]. Iran is a lower-middle-income country in EMRO that has been gearing up its attempt to achieve Sustainable Development Goals (SDGs) and tackle Noncommunicable Diseases (NCDs) [11]. However, the prevalence of obesity and its related complications in all age groups of Iranians is high and is increasing alarmingly [12–16].

Childhood obesity prevalence is increasing rapidly in Iran. A meta-analysis of 107 studies conducted on Iranian children estimated the prevalence of childhood overweight and obesity to be about 10.8% (95% CI, 10.2–11.4) and 5.1% (95% CI, 4.4–5.8), respectively [9]. Another meta-analysis that reviewed the prevalence of obesity in school-aged children estimated a similar rate (obesity prevalence: 5.82%; 95% CI, 5–6.66) [17]. The fourth phase of the “Childhood and Adolescence Surveillance and Prevention of Adult Non-communicable disease” survey (CASPIAN-IV) on 13,486 students in Iran reported overweight and obesity prevalence as 9.7 and 11.9%, respectively [18]. The covid-19 has deteriorated the conditions all over the world [19]. It is clear that childhood obesity prevalence has increased due to covid-19 in Iran. However, precise data is not yet available. This pandemic has raised the prevalence of childhood obesity and put several barriers to its control. Therefore, childhood obesity control should receive more attention and needs more innovative strategies in these years [20, 21].

Obviously, childhood obesity results from a multifactorial derangement of individual and social factors. This gives prominence to multidisciplinary approaches and interventions in tackling the growing prevalence of childhood obesity [4, 6, 22]. Several valuable efforts are underway to combat childhood obesity in Iran [23–25]; however, the alarming increase in this health problem highlights the need for more effective actions. Several gaps have been distinguished in the path that must be addressed to leverage the whole government and society approach.

Engaging the stakeholders and experts in the policy-making process is considered a significant determinant of effective interventions [26, 27]. They deeply know the context and can recognize the most effective strategies to battle childhood obesity. What is more, they bring about a comprehensive insight into the feasibility and stability of these strategies. The Delphi method is a repetitive process designed to understand conflicting issues better and help achieve consensus through controlled feedback [28]. During the Delphi process, stakeholders will be more sensitized about this health crisis, giving them a more profound sense of ownership of prioritized policies. The policy dialogue is another widely-accepted method in policymaking. It will sensitize stakeholders to this health issue and let the policy-makers listen to all stakeholders’ comments. This will result in more practical policies which are acceptable. All the mentioned issues will increase the chance of successful future implementations [29, 30].

Based on what was mentioned above, the current study aims to assist policy-makers in adapting practical and feasible policies to combat childhood obesity. It applied a mixed Delphi and policy dialogue approach to maximize the involvement of experts and stakeholders in exploring and prioritizing policy options for controlling childhood obesity in Iran.

## Methods

### Setting

**Fig. 1:**
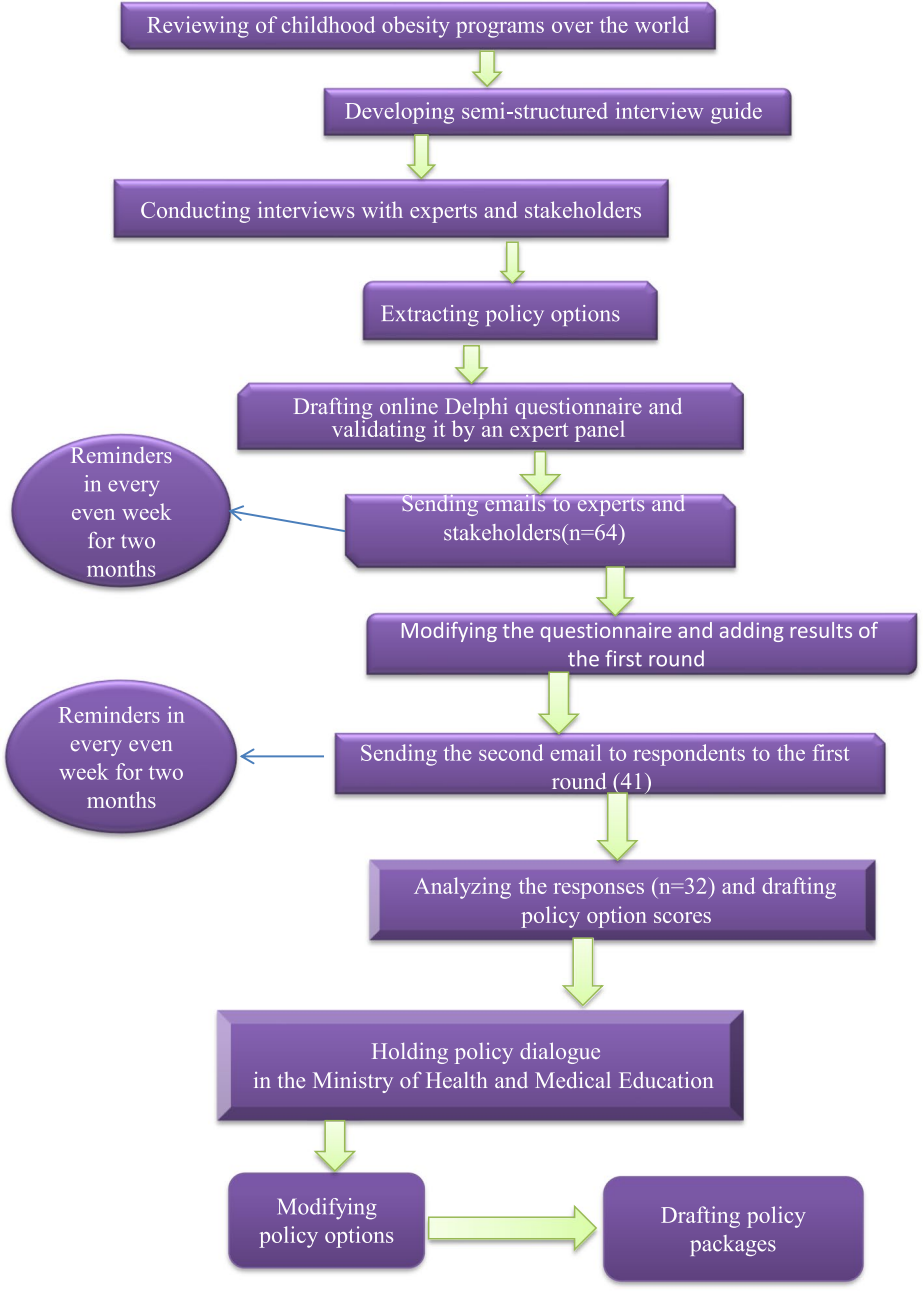
Steps to prioritizing policy options for childhood obesity control in Iran

This is part of a mixed-method prospective policy analysis to investigate attempts made by Iranian authorities to control childhood obesity and propose the required modifications. This part of the study was performed to set priority policies for preventing childhood obesity in Iran. This mixed Delphi and policy dialogue study is organized into three Delphi phases, followed by a policy dialogue. The graphical abstract of our study is provided in Fig. 1. It started with a comprehensive review followed by in-depth interviews and two rounds of an online Delphi survey from July 2021 to September 2021. In the fourth phase, 22 main stakeholders were invited to a policy dialogue session to discuss the policy options and draft a policy package.

#### Round 1: development and validation of the questionnaire

As pointed out in the literature, the first round of a classic Delphi approach generates ideas through interviews, focus group discussions, or open-ended questionnaires [28]. Therefore, the first step of the current Delphi study was interviewing experts and primary stakeholders. First, we have done a comprehensive review of suggested options in the literature or the policies implemented in countries that successfully combat childhood obesity. The interview guide was drafted based on this comprehensive review. Moreover, the guide was validated through pilot interviews with academic members specialized in community nutrition, health policy, and health education. As mentioned earlier, this study is a part of a more comprehensive survey. As a result, the interviews were comprehensive enough to investigate the main facilitator**s** and barriers to childhood obesity control in Iran. The characteristic of the interviewees is presented in Table 1. Purposive sampling and snowballing techniques were applied in this study phase, leading to 30 in-depth interviews before saturation. They were members of state organizations, academics specialized in different majors related to childhood obesity control, food industry managers, member of non-profit organizations, and international organizations. Key-informed persons from Ministry of Health and Medical Education, ministry of education, Secretariat of the Supreme Council for Health and Food Security, Food and Drug Administration, Municipality, Ministry of Interior and Islamic Republic of Iran Broadcasting were involved in all phases of this study.

**Table 1:** Characteristics of participants in Rounds 2 and 3 of Delphi

Participant’s characteristics		In-depth interview	Round 2				Round 3			Policy dialogue
			Invited to Round 2	Participated in Round 2			Participated in Round 3			
				number	percent	response rate	number	percent	response rate	
Specialty	Community Nutrition	6	9	9	22	100	9	28	100	7
	Clinical Nutrition	–	4	4	10	100	2	6	50	2
	Food policy	–	3	3	7	100	3	9	100	3
	Food science and technology	5	2	1	2	50	1	3	100	–
	General medicine	2	4	4	10	100	2	6	50	–
	Pediatrics	3	7	5	12	71	3	9	60	2
	Sociology	2	3	3	7	100	3	9	100	1
	Epidemiology, social science	4	8	2	5	25	1	3	50	2
	Health economy	1	3	1	2	33	1	3	100	–
	Health policy	–	4	0	0	0	–	–	–	1
	Health management	1	5	4	10	80	4	13	100	–
	Health education and promotion	–	3	1	2	33	1	3	100	–
	Physical education	1	2	1	2	50	1	3	100	2
	Other	5	7	3	7	43	2	6	67	2
	Total	30	64	41	100	64	32	100	78	22
Organization	Academic	11	35	22	54	63	19	59	86	10
	State organization	14	22	13	32	59	8	25	62	10
	Non-profit organization	1	4	4	10	100	3	9	75	–
	International organization	2	3	2	5	67	2	6	100	2
	Food industry	2	–	–	–	–	–	–	–	–
	Total	30	64	41	100	64	32	100	78	22

In these interviews, the participants were asked about the effective policies to control this problem in Iran. Based on their answers, the researchers completed the list of presumed effective policy options to combat childhood obesity in Iran. Then a questionnaire was developed to rate this policy option by stakeholders. The questionnaire was sent to 6 experts to evaluate its content validity. They specialized in community nutrition (2 experts), health policy, pediatrics, health education, and health promotion. They assessed the constructs of policy options and the priority criteria and checked the coherence and comprehensibility of all parts of the questionnaire. After covering the proposed modification, they confirmed the validity of the questionnaire.

#### Round 2: Likert scoring of policy options

Participants in Rounds 2 and 3 were recruited via purposive sampling. They represented three perspectives: policy-makers at different levels, presidents of various organizations who would implement potential policy options, and academics who had experience in this field to maximize the spread of opinions. There is no consensus on sufficient sample size for Delphi; however, existing literature suggested a purposive sampling of various stakeholders [31–34]. Some participants in the first round who had a commercial conflict of interest with the proposed policy (*n* = 3) were not invited to participate in n Rounds 2 and 3. Several new participants were added in Round 1 (*n* = 31) based on experts’ suggestions. Characteristics of the participants in Rounds 2 and 3 are shown in Table 1.

The panelists were asked to rate each policy option using a five-point Likert scale in seven constructs. A full description of the criteria for priority-setting used in this study is presented in the supplementary Table 1. A full description of the study’s protocol, including explaining the constructs and links to online questionnaires, was mailed to the participants. The panelists were asked to add their policy options, suggest other panelists, or point out any other suggestions.

Individual emails were sent to all participants in July 2021. They were given a maximum of 2 months to answer the questionnaire in R2. Reminders were sent at four-night intervals via the “what’s up” App and emails to those who had not responded.

#### Round 3: rescoring of policy options

In this round, the panelists were asked to re-rank policy options. Some options were added to the questionnaire based on the panelists’ suggestions in Round 2. The median and IQR of allocated scores given to each policy option in Round2 were pointed out in the online questionnaire of Round 3. An email containing a description of this phase and a link to the modified questionnaire was sent to the panelists who answered the questionnaires in Round 2 in October 2020. The reminders were sent every fourth night, and this round was closed after 2 months. As a consensus was achieved in almost all options in this round, the responses to Round 3 were analyzed to prioritize the options and the level of consensus.

### Policy dialogue

The policy dialogue session was held in the Nutrition Group Office of the Ministry of Health and Medical Education**.** The advocacy collation framework from the early stage of this study was applied to enhance the chance of a feasible and acceptable policy package. Twenty-two stakeholders, including scientists (*n* = 11) and executives from several organizations (n = 11), were invited. Only one person which was an academic member did not take part in the policy dialogue. As the prioritized policies need the collaboration of several ministries, the researchers decided to invite them to a policy dialogue session to review the policy options and design the policy package. The stakeholders were asked to prioritize five policy options that are the most effective policies in combatting childhood obesity in Iran. Moreover, they assisted in classifying policy options and producing a policy package draft.

### Data analysis

A draft of policy options was made after our comprehensive review. After each interview, the recordings of interview were transcribed verbatim and were perused several times to catch as many policy options as possible. Thematic analysis was conducted as recommended by qualitative research methods [35, 36]. Then, we modified our interview guide and the draft of policy options before the next interview. Interviews were continued until at least one stakeholder in each discipline was involved and new interviews add no new ideas.

The quantitative summary of responses given in Rounds 2 and 3, including the frequency, median, mean discerption index, and IQR of each option, were analyzed using Excel (Microsoft Excel Windows10). An IQR < 1 in Round 3 was used to indicate consensus for each policy option, and the highest median score and the lowest dispersion were considered to define policy options’ priorities. It was found that some effective options were not prioritized because of their low scores for the acceptance construct or low scores in other constructs. In this line, the median scores of policy options in each construct are provided in Table 3 to clarify the effective policy options that are not acceptable.

## Results

This study reviewed the literature on CO control to extract policy options and develop an interview guide. After validation of the guide, we interviewed 30 stakeholders in CO control. We applied the results of our comprehensive review to complete our policy options and validate them through ongoing interviews. The researchers introduced 27 policy options (Supplementary Table 2) and asked the stakeholders to rank them using seven criteria (Supplementary Table 1) on a five-level Likert scale. Sixty-four stakeholders were invited to rate policy options in two rounds. The participants were experts from universities and members of state, non-profit and international organizations. Totally, 41 experts participated in Round 2 (64% response rate), and 32 experts took part in Round 3 (78% response rate). Respondents from non-profit organizations showed the highest response rate (100%), and the response rate from other organizations was similar. A brief description of the participants in all parts of our study is shown in Table 1.

The scores of each policy option are provided in Table 2. Healthy schools, educating on appropriate supplementary feeding, and creating a healthy environment in kindergarten and other childcare centers received the highest scores. There was also consensus on these policies (IRQ < 0.7), with over 90% of the experts giving a score of 3.5 or over to these three options. The stakeholders reached a consensus for almost all policy options in Round 3. The stability of scores in Round 3 was high, with the SD/mean of below 0.3 for all policy options.

**Table 2:** Scoring of policy options to combat childhood obesity in Iran by stakeholders

policy options	Round 2		Round 3					
	med	IQR	med	IQR	Mean	SD	SD/mean	percent of respondents who scored > 3.5
The healthy school (healthy nutrition, physical activity, and health education)	4.05	0.67	4.48	0.65	4.36	0.55	0.13	94
Educating on appropriate supplementary feeding	4.14	1	4.17	0.65	4.15	0.55	0.13	91
Creating a healthy environment in kindergartens and other child-caring centers	*	*	4.14	0.68	4.17	0.6	0.14	91
Increasing access to physical activity facilities with a priority for deprived areas	*	*	4.02	1.09	3.89	0.6	0.15	69
Educating on healthy lifestyles in mass media	4	0.76	4.1	0.73	3.98	0.66	0.17	78
Developing a guideline on “nutrition, physical activity, and children’s lifestyle.”	3.9	0.71	4	0.77	4	0.67	0.17	81
More support for breastfeeding	4.19	0.95	3.98	0.77	3.98	0.57	0.14	84
Increasing the involvement of stakeholders in the policy-making process	3.62	0.81	3.95	0.87	3.92	0.57	0.15	78
Enhancing advertising control	3.67	0.67	3.95	0.88	3.89	0.66	0.17	72
Enhancing pregnancy cares	4	0.67	3.95	0.94	3.99	0.66	0.17	75
Advocacy for childhood obesity control	4.05	0.62	3.93	0.76	3.84	0.65	0.17	75
Increasing intersectional collaboration for better implementation of the policies	4	0.86	3.9	0.51	3.89	0.56	0.14	81
Increasing consultation and collaboration with international organizations	*	*	3.9	0.85	3.86	0.6	0.16	72
Modifying and better implementing the ratified policies	3.8	1	3.89	0.67	3.76	0.57	0.15	75
Providing better health care in PHC with a priority given to prevention	4.1	0.67	3.88	0.92	4.01	0.66	0.16	81
Enhancing academic education related to obesity in medical schools	3.95	0.9	3.86	0.71	3.92	0.69	0.18	84
Social marketing to combat childhood obesity	3.86	0.57	3.86	0.83	3.8	0.68	0.18	78
Developing, ratifying, and notifying the policy package “Enhancement of Nutrition and Physical Activity in Children.”	3.76	0.9	3.86	0.93	3.8	0.67	0.18	69
Subsidizing healthy foods	3.71	0.95	3.81	0.79	3.74	0.8	0.21	72
Improving food labeling	3.81	1	3.81	0.98	3.74	0.67	0.18	59
Ensuring weight management cares	3.81	0.71	3.79	0.77	3.78	0.58	0.15	75
Taxation on unhealthy foods	3.57	1.09	3.76	1.07	3.79	0.75	0.29	72
Modifying food baskets of supporting institutions	3.76	0.86	3.63	1.15	3.62	0.82	0.23	59
Food reformulation	3.48	1.19	3.57	0.81	3.43	0.67	0.19	53
Modifying agricultural and commerce policies to provide nutrients rather than the sole energy	3.61	0.81	3.57	1.1	3.61	0.75	0.21	56
A conditional cash transfer to families	3.1	3.76	3.55	0.96	3.32	0.75	0.23	50
Environmental reengineering to increase the possibility of physical activity	*	*	3.55	1.11	3.59	0.75	0.21	59

Options are sorted based on the Med of allocated scores in the third round of Delphi

A description of these policies is provided in Supplementary Table 2

*This option has been added in the third round of Delphi

Detailed scores of policy options in each criterion in Round 3 are provided in Table 3. The most effective policies given to the stakeholders were healthy school (featuring healthy nutrition, physical activity, and health education), creating a healthy environment in kindergartens and other child caring centers, educating on appropriate supplementary feeding, educating on healthy lifestyles in mass media, and social marketing to combat childhood obesity. All participants gave the healthy school a score of five in the effectiveness construct. “Increasing intersectional collaboration for better implementation and expanding the partnership of stakeholders in the policy-making” stands next to the healthy school. All other policy options were reported to be effective, with a score of four. All policy options were deemed to be relevant by the participants.

**Table 3:** Detailed scores of policy options in Round 3 of Delphi for each criterion

policy options	Total score	Effectiveness	Relevance	Feasibility	Acceptable cost	Acceptability to			health equity promotion	Easy monitoring
						politicians	society	executives		
The healthy school (healthy nutrition, physical activity, and health education)	4.48	5	5	4	4	4	5	5	5	4
Creating a healthy environment in kindergartens and other child-caring centers	4.14	5	5	4	4	4	5	4	4.5	4
Educating on appropriate supplementary feeding	4.17	4	5	4	4	4	4	4	5	4
Educating on healthy lifestyles in mass media	4.10	4	5	4	3.5	3.5	4	4	4.5	4
Increasing access to physical activity facilities with a priority for deprived areas	4.02	4	4.5	3.5	3	4	4	4	5	4
Developing a guideline on “nutrition, physical activity, and children’s lifestyle.”	4	4	4.5	4	4	4	4	4	4	4
More support for breastfeeding	3.98	4	5	4	4	4	4	4	4	4
Enhancing pregnancy cares	3.95	4	4	4	4	4	4	4	4	4
Increasing the involvement of stakeholders in the policymaking process	3.95	4.5	5	3.5	4	4	4	4	4	3
Enhancing the control of advertising	3.95	4	5	3	3	3	4	4	4	4
Advocacy for childhood obesity control	3.93	4	4	3	4	3	4	4	4	3
Increasing consultation and collaboration with an international organization	3.9	4	4	4	4	3	4	4	4	4
Increasing intersectional collaboration for better implementation of the policies	3.9	4.5	4	3	4	3.5	4	4	4	3.5
Providing better health care in PHC with a priority given to prevention	3.88	4	5	3.5	4	4	4	4	4.5	4
Modifying and better implementing the ratified policies	3.86	4	4	4	4	4	4	4	4	3.5
Enhancing academic education related to obesity in medical schools	3.86	4	4	4	4	4	4	4	4	4
Developing, ratifying, and notifying the policy package “Enhancement of Nutrition and Physical Activity in Children.”	3.86	4	4	3	4	4	4	4	4	3.5
Social marketing to combat childhood obesity	3.86	5	4	4	4	3.5	4	4	4	3
Improving food labeling	3.81	4	4	4	4	4	4	3	4	4
Subsidizing healthy foods	3.81	4	4	3	3	3	4	4	4.5	3
Ensuring weight management cares	3.79	4	4	3	3	3	4	3.5	4.5	4
Taxation on unhealthy foods	3.76	4	4	3	4	3	4	4	4	3
Modifying food baskets of supporting institutions	3.6	4	4	4	3	3	4	3.5	4	4
Modifying agricultural and commerce policies to provide nutrients rather than the sole energy	3.57	4	4	3	3	3	4	3	4.5	3
Food reformulation	3.57	4	4	3	3	3	4	4	4	3
A conditional cash transfer to families	3.55	4	4	3	3	3	3	4	4	3
Environmental reengineering to increase the possibility of physical activity	3.55	4	4	3	3	3	4	4	4	3.5

Options are sorted based on the Med of the total score

Results are presented as the Med of scores

A description of these policies is provided in Supplementary Table 2

A description of scoring criteria is provided in Supplementary Table 1

Concerning feasibility and cost, all options were found feasible with an acceptable cost as most of them received a four score, and none received a score under 3. The participants rated all options as acceptable.

Considering health equity, the participants believed that healthy schools, education on appropriate supplementary feeding, and increasing access to physical activity facilities with a priority on deprived areas are the most equity-oriented options. Healthy schools were considered the most effective way to promote health equity. Creating a healthy environment in kindergartens and other child caring centers, educating on healthy lifestyle in mass media, providing health care in PHC with a priority given to prevention, subsidizing healthy foods, ensuring weight management cares, and modifying agricultural and commerce policies stand just after them. Policy options were not mainly different in scale of easy monitoring of our stakeholders’ views.

Twenty-two stakeholders took part in the Policy Dialogue held in the Ministry of Health and Medical Education: Ten scientists and 10 executives from organizations involved in childhood obesity control, and 2 members of international organizations. They confirmed that healthy schools are the most effective and feasible policy to control the issue. However, they believed that policies that increase families’ access to healthy foods, mainly subsidizing healthy foods, should be applied, although they are not easy to ratify and implement. The participants also believed that healthy schools, creating a healthy environment in kindergartens and other child care centers, subsidizing healthy foods, educating on healthy lifestyles in mass media, and increasing access to physical activity facilities with a priority given to deprived areas are the most effective policies in controlling childhood obesity. The “Healthy Schools” policy was suggested as the most effective policy by 53% of the stakeholders, and others believed this is the second most important. Participants in the policy dialogue rated creating a healthy environment in kindergartens and other childcare centers as the second most important policy. They believed that subsidizing healthy foods should be considered the third effective policy. This was the only option for which the position changed compared to Delphi’s results. A draft of a policy package to combat childhood obesity in Iran was developed in this session; the list of the policies is provided in Table 4.

**Table 4:** The proposed policy packages to combat childhood obesity in IR Iran

Policy package	Policy options
Healthy Families	Educating on healthy lifestyles in mass media
	Educating on appropriate supplementary feeding
	More support for breastfeeding
	Social marketing to combat childhood obesity
	A conditional cash transfer to families
Healthy schools, kindergartens, and other healthcare centers	Improving the school milk program
	Improving a healthy breakfast program
	Controlling food markets in schools and neighborhoods
	Educating on healthy lifestyles in school curricula
	Increasing the possibility of walking to schools
	Increasing physical activity facilities in schools and parks close to schools
	Adding at least 3 hours of physical education to school curricula
	Employing an adequately-trained exercising coach
	Screening the weight and height of children and referring and monitoring the malnourished ones
Increasing the availability of healthy foods	Enhancing the control of advertising
	Subsidizing healthy foods
	Taxation on unhealthy foods
	Food reformulation
	Modifying food baskets of supporting institutions
	Modifying agricultural and commerce policies to provide nutrients rather than the sole energy
Providing an active environment	Increasing access to physical activity facilities with a priority for deprived areas
	Environmental reengineering to increase the possibility of physical activity
	Increasing the possibility of physical activity for girls and women
	Modifying amusement parks to health-oriented centers
Improving health cares	Developing a guideline on “nutrition, physical activity, and children’s lifestyle.”
	Enhancing pregnancy cares
	Providing better health care in PHC with a priority on prevention
	Enhancing academic education related to obesity in medical schools
	Ensuring weight management cares
Propper governance and leadership	Increasing the involvement of stakeholders in policymaking
	Advocacy for childhood obesity control
	Increasing consultation and collaboration with international organizations
	Increasing intersectional collaboration for better implementation of the policies
	Modifying and better implementing the ratified policies
	Developing, ratifying, and notifying the policy package “Enhancement of Nutrition and Physical Activity in Children.”

## Discussion

This is a mixed Delphi and Policy Dialogue study which includes a three-round Delphi study followed by a Policy Dialogue to prioritize policy options for controlling CO in Iran. Stakeholders from various specialties and organizations took part in this study. Healthy schools, educating on appropriate supplementary feeding, and creating a healthy environment in kindergartens and other child care centers were the most prioritized policy options in the Delphi phase of the study. These options also received high scores in different criteria, including effectiveness, health equity, feasibility, and acceptance. Stakeholders in the Policy Dialogue session confirmed the priority of healthy schools. Moreover, they emphasized the priority of policies that increase the accessibility of healthy foods, mainly through subsidizing healthy foods. The participants in this section developed an initial policy package draft to combat childhood obesity in Iran.

As evident in Table 3, some policy options are considered effective but not acceptable or feasible. It is critical to consider this fact if we aim to control childhood obesity propitiously. Some effective policies are not considered a priority by the panelists because they believe there is a lot of conflict of interest about them, or they cost more than policymakers are willing to pay [37]. We must more persuasively advocate for policy-makers and even street-level bureaucrats such as school administrators, food producers, or health center managers to accept these options. However, in the policy dialogue section, the stakeholders emphasize the importance of these policy options, mainly subsidizing healthy foods.

The participating stakeholders mainly insist that the most effective policy to control this issue is “healthy schools.” This policy option is one of the central parts of WHO Ending Childhood Obesity (ECHO) [38], whose implementation in several countries has successfully controlled obesity [39]. It is at the focal point of efforts for childhood obesity control because children spend most of their time in school. It could cover almost all children as over 90% of children go to schools in most countries, including Iran. Schools are commonly considered the place to learn for future life; therefore, children and their parents are ready to be educated and have a healthy lifestyle [39]. Several attempts have been made to make Iranian schools healthier, including controlling food provision and educating about healthy lifestyles. However, interviewed stakeholders in the first phase of our study believed that these measures have not been implemented effectively in our country. Moreover, the covid-19 pandemic lockdowns have deteriorated the condition. Moreover, our participants believed in a comprehensive school program which was defined in Table 4. This program is compatible with the characteristic of effective school intervention described in a systematic review and meta-analysis by Singhal and his colleagues [40].

The effect of the Covid-19 pandemic on obesity control, particularly childhood obesity control, should be considered. The covid-19 pandemic intensifies the importance of obesity control in all age groups globally. Several studies have demonstrated a mutual association between obesity and the covid-19 pandemic. It has been thoroughly proven that obesity disturbs the immune response to several infections [1]. Several studies have shown that covid-19 infection results in more deadly consequences in obese patients [41–43]. Moreover, the obesity pandemic has accelerated during this pandemic. Several lockdowns and other restrictions decrease physical activities in all age groups, and children are affected mainly by closing schools. Unfortunately, most interventions to combat childhood obesity are settled in schools, increasing the importance of focusing on childhood vaccination and opening schools [3, 20, 21, 44].

Stakeholders in this study insist on implementing policies that can increase families’ access to healthy foods. They believe that financial measures would affect families’ food choices. They stand firm that subsiding policies should be modified to increase access to healthy foods. In accordance with our findings, a comprehensive study has shown that low- and middle-income countries mainly subsidize food items that provide energy. These fiscal policies were significantly associated with the body weights of their population [45]. Unfortunately, the targeted subsidies policy in Iran reduced the intake of meat, dairy, fruit, and vegetables [46]. This will increase the risk of obesity particularly in lower socioeconomic groups.

Several studies have been conducted on weight control obstacles from the point of view of children or adults in Iran [47–49], But only one study has examined barriers and facilitators from the perspective of all stakeholders recently. This study identified barriers and facilitators through in-depth interviews and then ranked them through two Delphi rounds. Although most of the stakeholders were from one province of Iran, they perfectly depicted the weak points of CO control at the national level. They classified barriers and facilitators into three levels: individual, executive, and structural. The most important factors were placed at the structural level. Fortunately, almost all of the barriers cited in their study are addressed in our policy package, including top-down policymaking process; poor implementation, and monitoring of policies; high economic and physical access to unhealthy foods, low access to physical activity facilities, unhealthy school environment, and low parental and child awareness [50].

Our study’s strength is the inclusion of policy-makers in diverse fields related to childhood obesity, which is integral to the successful ratification and implementation of prioritized policy options [51]. This study applies an advocacy collation framework [52] and an evidence-informed policy-making approach [53], increasing the chance of feasible and acceptable policies. The researchers eagerly tried to involve all stakeholders in all the rounds of this study to increase the acceptability of the policies and the chances of better implementation [51].

Our three-round Delphi process was another strength of our study. Moreover, we began with a qualitative part, which is an advantage of our method. The literature defines that the classic Delphi consists of four rounds. It would be better to start with a qualitative interview phase or a focus group discussion. More recent studies have shown that two scoring rounds are more efficient than a single round, and repetition will cause fatigue and decrease participation and precision [28, 54]. We confirmed our results by holding a policy dialogue phase [29, 30], another strength of our study.

The Delphi method is susceptible to researcher and subject bias because the individuals who are more affected by decisions are more interested in participating in the study. Moreover, as the participants are informed about the majority’s response, they may change their answers in line with the majority. However, it has been perceived as an advantage that brings participants to group consensus [28]. The panelists of this study took part in a deep individual interview and two rounds of online surveys without any proximity or face-to-face meeting, which allowed them to think and not be affected by the dominant view [55, 56].

However, this is a qualitative study, and participants in this study scored policy options subjectively. Although the participants were closely engaged with CO control, the results of this study should be confirmed by a more validated type of study. It is the first step of this long journey and the next step should be assessing the real-world effectiveness of this policy package in a pilot study. Moreover, cost-effective analyses of the proposed policies could provide convincing evidence for policymakers in this field.

Obesity control is a central part of Iranian health-enhancing priorities. Several attempts have been made to combat childhood obesity in Iran, and several studies have assessed the effectiveness of the known strategies. However, the rising trend of this problem indicates the urgent need to act more effectively. This study addressed the prioritized strategies that should be ratified and implemented to combat childhood obesity in Iran with the comprehensive views of diverse stakeholders. Moreover, it revealed that some effective strategies are not acceptable to politicians, society, or street-level bureaucrats, which indicates a need for effective and professional advocacy. The prioritized policy options in this study will increase the chance of healthy weight for children and enhance children’s health in all aspects. Additionally, they will affect the health of all the members of society through integrative policy options.

## Conclusions

Obesity control is an integral part of controlling non-communicable diseases, and all countries should implement a proper obesity management strategy to achieve SDH goals. Effective control of CO is an essential component of obesity control. Several attempts have been made to combat childhood obesity in Iran. However, the alarming increase in this health problem clarifies the need for more effective measures. Several gaps have been distinguished in this path that must be addressed to leverage the whole government and society approach in this field. Prioritized policies in the current study can help policy-makers to enhance their performance. To do so, the researchers used the advocacy collation framework and the evidence-informed policy-making approach, which increased the acceptability and feasibility of the developed policy package. The next step will be impressible advocacy to ratify and implement these policies.

## Supplementary Information

**Additional file 1: Supplementary Table 1.** Description of priority-setting criteria used in this study. **Supplementary Table 2.** Brief description of policy options ranked in this study.

## Data Availability

For confidentiality, we cannot give access to crude interviews. Other data, including coded segments, are available on request through an email to A.T.
