# Supplementary material for: Setting and prioritizing evidence-informed policies to control childhood obesity in Iran: a mixed Delphi and policy dialogue approach
Source: BMC Pediatr. 2022 Dec 20;22:724. doi: 10.1186/s12887-022-03796-z (PMC9763081; doi:10.1186/s12887-022-03796-z)
Supplement: Supplementary file 1 — Additional file 1: Supplementary Table 1. Description of priority-setting criteria used in this study. Supplementary Table 2. Brief description of policy options ranked in this study. [file 12887_2022_3796_MOESM1_ESM.docx]

Supplementary tables:

| Supplementary Table 1. Description of priority-setting criteria used in this study | |
| --- | --- |
| Description | Criterion |
| The extent to which the policy option affects childhood obesity. | Effectiveness |
| The extent to which the policy option is relevant to childhood obesity control in our society. Is it pertinent to our context? | Relevance |
| Is it realistic and possible to implement this policy option in our society? Are there any technical, workforce, or legal barriers? | Feasibility |
| Is the cost of this option acceptable and affordable to policymakers? | Acceptable cost |
| The extent to which policymakers accept ratifying the policy option. | Acceptability to politicians |
| Is the option acceptable to the general population? | Acceptability to society |
| The extent to which the policy option is likely to be judged as legitimate and then accepted, adopted, and implemented by executives and street-level bureaucrats. | Acceptability to executives |
| Will the policy option decrease inequality by reducing the differences in health indexes among different socioeconomic groups? | Health equity promotion |
| Are process mapping and impact evaluation of the policy option simple and easily managed? | Easy monitoring |

| Supplementary Table2. Brief description of policy options ranked in this study | | |
| --- | --- | --- |
| Row | Policy option | Description |
| 1 | Modifying and better implementing the ratified policies | This option emphasizes the fact that several effective policies have been ratified. However, they are not successfully implemented because of ignoring the context and structure of the society. Therefore, instead of ratifying new policies, we needed to investigate the cause of failure and modify and implement the existing policies. |
| 2 | Increasing intersectional collaboration for better implementation of the policies | This option supports the necessity to increase intersectional collaboration among all organizations involved in childhood obesity intervention. It will reduce duplication and will boost effective implementation. |
| 3 | A healthy school (healthy nutrition, physical activity, and health education) | This is a series of targeted actions in schools, including providing healthy foods, controlling school cafes, enhancing health education, increasing physical activity, facilitating walking to school, nutritional screening, and referring overweight and obese children to health centers. |
| 4 | Creating a healthy environment in kindergartens and other child caring centers | This policy option consists of profound control of all child care centers, including kindergartens, to provide healthy foods to children and increase the opportunities for physical activity. Providing health education to children and parents and nutritional screenings are other vital policy options. |
| 5 | Educating on healthy lifestyle in mass media | This policy includes increasing health education on mass media and improving strategies and approaches to change society’s practices. |
| 6 | Strengthening control on advertising | Clearing the definition of health disturbing foods which must not be advertised. Removing the penalty of their advertisements and more restricted control on advertising to children. |
| 7 | Improving food labeling | Improving food labeling to be easy to understand and guide parents and children to healthier choices. Increasing public skills to choose foods by reading and understanding these labels. |
| 8 | Food reformulation | This option includes reformulating foods to healthier ones with lower fat, sugar, and salt without increasing their prices, particularly children’s foods. |
| 9 | Taxation on unhealthy foods | Codifying, ratifying, and implementing taxation on unhealthy food with a distinct definition of unhealthy foods and taxation methods. Earmarking these taxes to prevent health problems, particularly obesity prevention programs. |
| 10 | Subsidizing healthy foods | Allocating loans and financial incentives to producers of healthy foods to provide low-price healthy foods to the community |
| 11 | A conditional cash transfer to families | Conditioning cash transfers to families participating in health education programs and children's weight management |
| 12 | Modifying food baskets of supporting institutions | It is adjusting food baskets of charities to more nutrient-dense ones, which lessen the risk of obesity. Better supervision on distribution to ensure it is fed to at-risk members of families. |
| 13 | Modifying agricultural and commerce policies to provide nutrients rather than the sole energy | It modifies agricultural and commerce policies to provide nutrient-dense, high-protein foods such as beans, dairies, fruits, and vegetables rather than sugar and oilseeds. |
| 14 | Developing, ratifying, and notifying the policy package "Enhancement of Nutrition and Physical Activity in Children." | It provides involvement of all stakeholders in codifying, ratifying, and implementing a policy package that aims to promote healthy nutrition and physical activity. |
| 15 | Increasing collaboration with civil societies | This policy increases collaboration with civil societies in decision and policymaking and assists their capacities in implementing the policies. |
| 16 | Increasing consultation and collaboration with international organizations | Taking advantage of consulting with international organizations’ financial and scientific assistants. |
| 17 | Increasing the involvement of stakeholders in policymaking | Collaborating with all stakeholders, such as the food industries, universities, scientific associations, the Ministry of education, The Ministry of the Interior, etc... will lead to more efficient policies and increase their assistance in the implementation process. |
| 18 | Social marketing to combat childhood obesity | This is a long-term social marketing policy addressed to all society, particularly the parents, through social media, mass media, schools, mosques, and NGOs. It will increase public interest in childhood obesity and make it a public demand. |
| 19 | Advocacy for childhood obesity control | This policy advocates the policymakers to sensitize them about childhood obesity. |
| 20 | Increasing the access to physical activity facilities with a priority for deprived areas | Supplying all schools and public parks with free physical activity equipment, particularly in deprived areas, improves physical activity safety in public places, especially for girls. |
| 21 | Environmental reengineering to increase the possibility of physical activity | Environmental reengineering to enhance public transfer rather than using cars. Increasing safety for children’s physical activity, mainly their walking or cycling to schools. |
| 22 | Enhancing pregnancy cares | Upgrading the pregnancy care policies to increase the importance of NCD prevention, particularly healthy weight gain and physical activities |
| 23 | More support for breastfeeding | Boosting breastfeeding support by increasing maternity leave, social marketing, providing breastfeeding places in public areas, controlling supplying infant formulas |
| 24 | Developing guidelines on "nutrition, physical activity, and children’s lifestyle." | Developing comprehensive guidelines on "nutrition, physical activity, and lifestyle of children" by the Ministry of Health in collaboration with nutrition, health, and medical schools |
| 25 | Educating on appropriate supplementary feeding | Increasing awareness of the society on appropriate supplementary feeding through mass media |
| 26 | Providing better health care in PHC with a priority to prevention | Educating health care providers to highlight obesity prevention. Emphasizing the screening of obese children and following their weight management plan through a multi-discipline team. |
| 27 | Enhancing academic education related to obesity in medical schools | It indicates the modification of academic medical education to emphasize screening and treating childhood obesity in all health disciplines. |
| 28 | Ensuring weight management cares | Codifying weight control guidelines and ensuring health cares for obesity prevention and treatment. Emphasizing the role of nutritional, psychological, and physical consultation in childhood obesity treatment. |
